# Dermoid Cyst of the Floor of the Mouth: Diagnostic Imaging Findings

**DOI:** 10.7759/cureus.2403

**Published:** 2018-04-02

**Authors:** Leonardo Giarraputo, Sergio Savastano, Emanuele D'Amore, Ugo Baciliero

**Affiliations:** 1 Radiologia, San Bortolo Hospital, Vicenza, ITA; 2 Anatomia Patologica, San Bortolo Hospital, Vicenza, ITA; 3 Chirurgia Maxillo Facciale, San Bortolo Hospital, Vicenza, ITA

**Keywords:** dermoid cyst, floor of the mouth, diagnostic imaging, ultrasound, computed tomography, mri

## Abstract

Dermoid cysts of the floor of the mouth are rare, accounting for 11% of all dermoid cysts in the head and neck region. We report a case of a dermoid cyst of the floor of the mouth in a 12-year-old boy investigated with ultrasonography, magnetic resonance imaging (MRI), and non-enhanced computed tomography (CT) scans. The lesion contained free calcified corpuscles (i.e., the “sack of marbles” sign) considered pathognomonic for a dermoid. Diagnostic imaging may allow diagnosis of a dermoid of the floor of the mouth and plays a pivotal role in depicting the anatomic location of a cyst, thus guiding the surgeon for an optimal surgical approach.

## Introduction

Cysts on the floor of the mouth are classified into three types: epidermoid cysts, dermoid cysts, and teratomatous cysts. Each type is lined by an epithelial layer, but each type exhibits different histologic patterns. Dermoid cysts contain skin appendages and cystic teratomas present tissue originating from all the germinal layers. Dermoid cysts of the head and neck are relatively rare, accounting for 7% of all dermoid cysts [1]. Dermoid cysts differ from epidermal or epidermoid cysts given that dermoid cysts are localized in the dermal layer and are formed by several mechanisms such as the occlusion of a sebaceous duct or entrapment (both congenital and post-traumatic) of epidermal cells. Histologically, epidermal cysts can be easily differentiated from dermoid cysts due to the absence of skin appendages [2-3].

Dermoid cysts are slow growing and painless masses; when large enough, they can cause dysphagia, dysphonia, and dyspnea [4]. Diagnostic imaging plays a pivoting role in the anatomic localization of the mass in the floor of the mouth and, therefore, helps in guiding surgical planning [5-7].

## Case presentation

**Figure 1:**
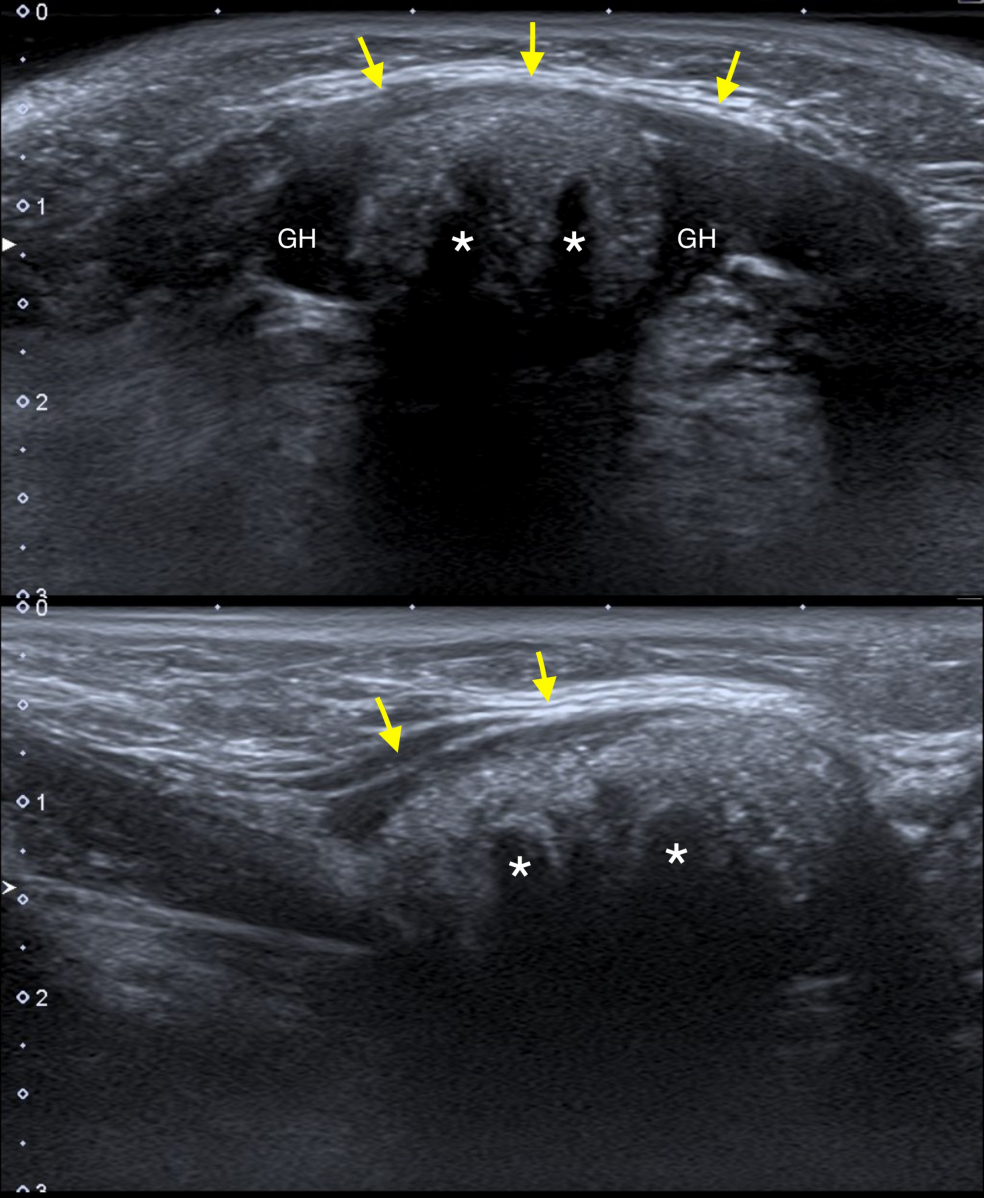
Submandibular ultrasonography Transverse (upper panel) and longitudinal (lower panel) scans show a hyperechoic lesion above the mylohyoid muscle (arrows) and between the geniohyoid muscles; highly reflective echoes with acoustic shadowing are appreciable (asterisks).

A 12-year-old boy was referred to the Department of Radiology of the San Bortolo Hospital (Vicenza, Italy) for a submandibular soft, painless mass. Ultrasonography showed an inhomogeneous hyperechoic mass, measuring 20 x 15 x 15 mm localized just above the mylohyoid muscle; intracystic highly hyperechoic corpuscles with shadowing were also noted (Figure 1).

**Figure 2:**
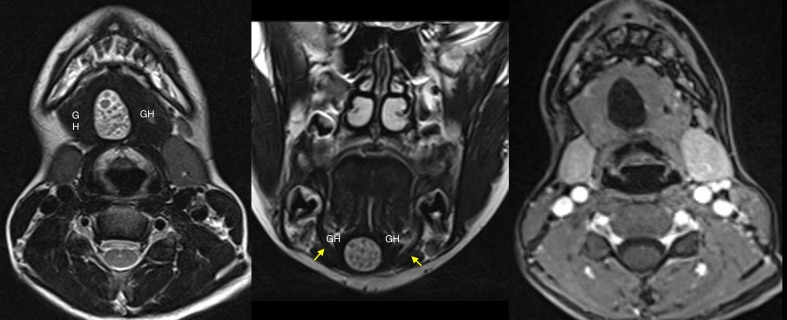
Magnetic resonance imaging (MRI) Axial T2-weighted-MRI (left panel) demonstrates a well-defined cystic mass between the geniohyoid muscles (GH); intracystic low-in-signal free bodies are also visualized. Coronal T2-weighted-MRI (middle panel) depicts the sublingual location of the cyst which lies above the mylohyoid muscle (arrows) between the GH. The cystic lesion does not enhance on post-contrast MRI (right panel); the intracystic corpuscles are faintly visible.

Magnetic resonance imaging (MRI) confirmed the location of the lesion above the mylohyoid muscle, between the geniohyoid muscles (Figure 2). The mass appeared low in signal on T1-weighted MRI and hyperintense on T2-weighted sequences. We also detected hypointense free intracystic corpuscles, which are more conspicuous on T2-weighted MR images. No enhancement was appreciable on post-contrast MRI (Figure 2). 

**Figure 3:**
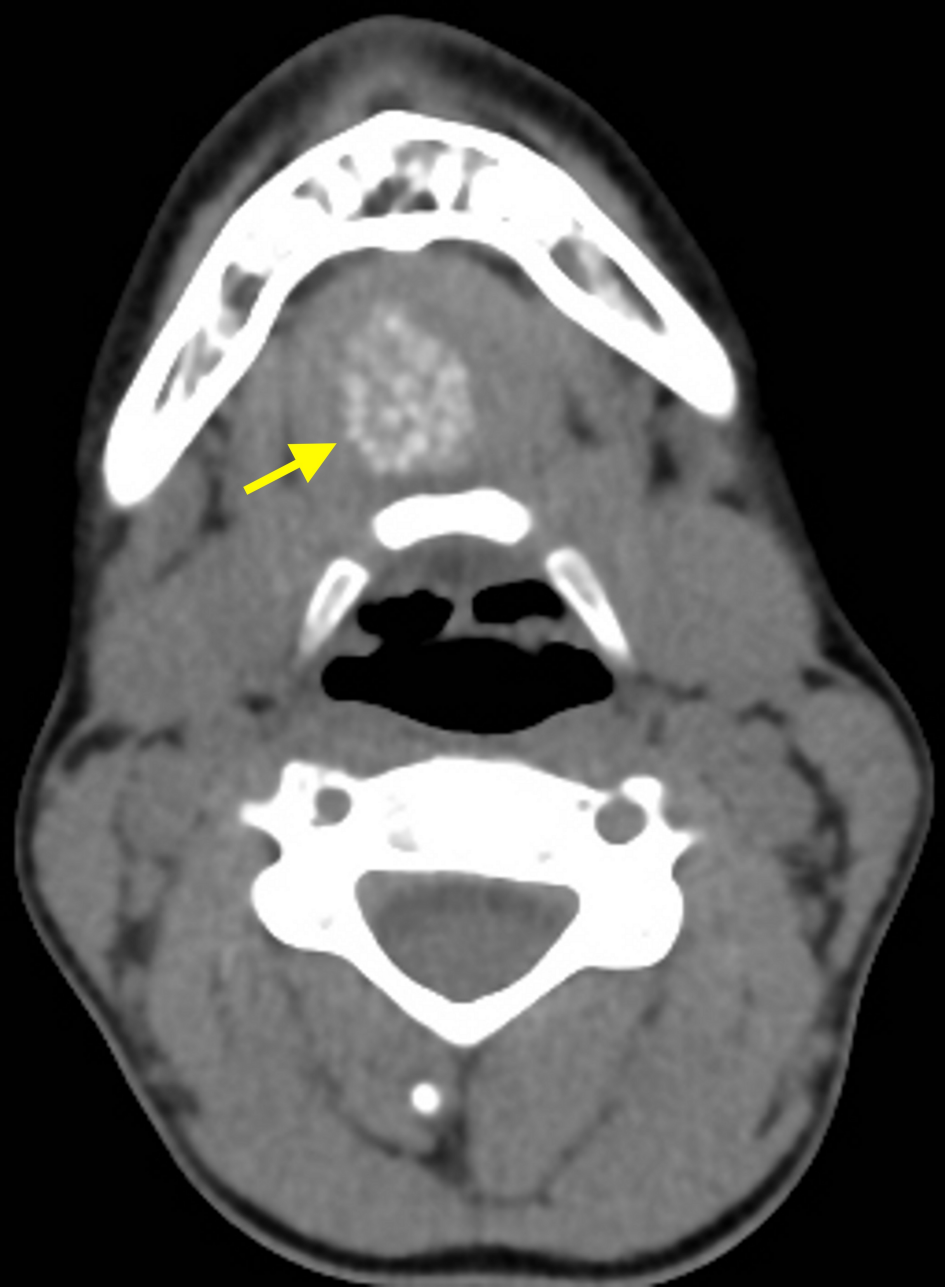
Computed tomography scan The intracystic free corpuscles are calcified (arrow) on non-enhanced computed tomography scan.

Intracystic corpuscles appeared calcified on non-enhanced computed tomography (CT) scans (Figure 3).

The patient underwent the surgical operation via a submandibular incision. The surgical specimen consisted of a cystic lesion with a 2.5-cm maximum diameter surrounded by a small amount of soft tissue.

**Figure 4:**
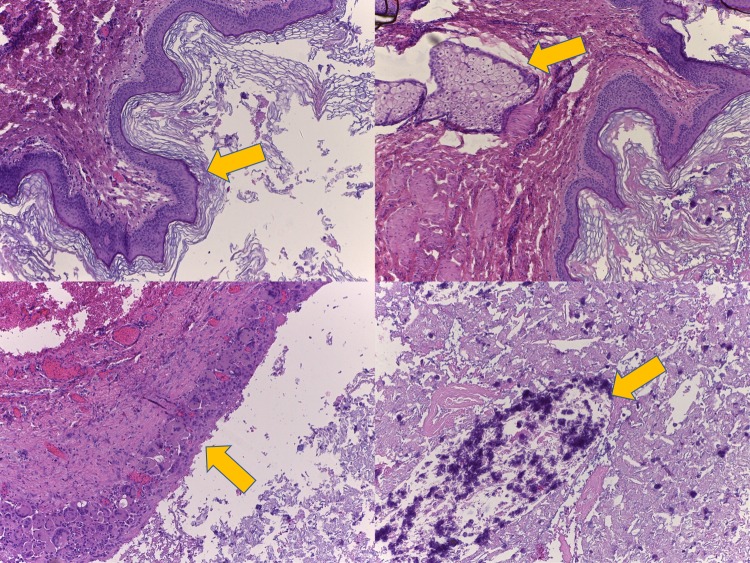
Histologic features The cyst is lined by keratinizing squamous epithelium (upper left panel) and contains a sebaceous gland in the wall (upper right panel). In the area of rupture, the epithelium is replaced by a granulomatous reaction (lower left panel). The desquamated keratin filling the cyst is partly calcified (lower right panel).

The histopathologic examination revealed a cyst lined by a keratinizing squamous epithelium, which was partly ruptured and replaced by a granulomatous reaction and contained adnexal structures in the wall (Figure 4).

## Discussion

Cysts on the floor of the mouth are classified into three categories: dermoid, epidermoid, and teratomatous cysts.

The mouth floor represents the second most common location of all dermoid cysts (approximately 11%) of the head and neck region, yet dermoid cysts are much more frequent there than epidermoid cysts [7]. With respect to the anatomical relationships with the mylohyoid muscle, dermoid cysts can be distinguished in sublingual, submental, and submandibular tissue, and each location requires a different surgical approach [5-7]. Dermoid cysts of the floor of the mouth are usually found in the midline, but they can also be laterally located as they grow [2].

Among imaging modalities, MRI is the gold standard for diagnosing cystic mass and depicting anatomy of the mouth floor given its excellent soft tissue contrast and multiplanar imaging capability [5,8].

Diagnostic imaging can reveal intracystic floating corpuscles (i.e., the “sack of marbles” sign) which are usually hypodense on CT scans, hyper/hypointense on T1-weighted MRI and hypointense on T2-weighted MRI scans because of their lipid contents, the presence of which is pathognomonic for a dermoid cyst [2,4,7]; the “marbles” can be calcified, as in the present case. Because of dense cystic fluid, a percutaneous fine needle aspiration may be not recommended [6].

Diagnostic imaging plays a pivotal role in the preoperative workup; the exact location of a cystic mass of the mouth floor is essential for determining the optimal surgical approach [5]. In our opinion, coronal T2-weighted MRI is mandatory to depict the relationship between a cystic lesion on one side and the geniohyoid and mylohyoid muscles on the other side [5].

Dermoid cysts of the floor of the mouth should be differentiated from other purely cystic and cyst-like lesions of the neck. Key differentiation may be suggested by a characteristic location or by enhancing the radiologic pattern after intravenous contrast medium administration.

Ranulas are retention cysts arising from a sublingual gland or accessory salivary glands and are rarely found in the midline. They can be simple or plunging in the submandibular space extending through a defect in the mylohyoid muscle (i.e., the boutonniere) or because it ruptures posteriorly. Evidence of a beak of the cyst towards the sublingual space (tail sign) is considered typical of ranulas [4,8]. However, a dermoid cyst entirely confined within the sublingual space cannot be distinguishable from a ranula by imaging findings alone [7].

Thyroglossal duct cysts are located in the midline or within 2 cm of the midline; most are detected at the level and below the hyoid bone, but they can be exceptionally discovered at the mouth floor [4,7,9]. Infection and cutaneous fistulization are usual complications of a thyroglossal duct cyst [7], but an uncomplicated cyst does not show a peculiar pattern on ultrasonography, CT or MRI.

Branchial cleft cysts are unilateral lesions that show different localization depending on the type of cyst [7-9]. First branchial cysts are close to the parotid gland, whereas second branchial cysts (the most common branchial cleft anomalies) can be located just anteriorly to the sternocleidomastoid muscle (Type-I), between the sternocleidomastoid muscle and the carotid space (Type-II), between internal and external carotid arteries and the pharynx (Type-III), and within the mucosal pharyngeal space (Type-IV). Vice versa, the third and fourth branchial cleft anomalies are more caudal, anatomically related to the piriform sinus [9].

Cystic hygromas usually involve the posterior cervical space or the floor of the mouth cavity; they show an infiltrative growth irrespective of anatomic planes. Very large unilocular lymphangioma can spread within the mediastinum and the axilla. Because of their high proteinaceous content, they show fine intracystic echoes on sonography and high signal on T1-weighted MRI; sometimes an intracystic fluid-fluid level can be appreciable [7-9].

The diagnosis of an oral abscess is easily suspected in the appropriate clinical setting; diagnostic imaging reveals an uninoculated or multiloculated fluid collection with an enhanced rim after an intravenous contrast medium is administered [4,9]. Post-contrast CT scans and MRI allow diagnosis of a hemangioma, which appears as lobulated enhancing masses [4].

## Conclusions

Diagnostic imaging may help in the correct diagnosis of a dermoid cyst of the floor of mouth; the sack-of-marbles sign is characteristic for a dermoid. MRI is specifically useful in depicting the anatomic relationship of the dermoid cyst and muscles of the floor of the mouth, and, therefore, in guiding the surgeon to the more appropriate approach for surgical excision. Knowledge of both imaging patterns and typical location of another cystic lesion of the mouth and neck may help in the differential diagnosis.
